# When the diagnosis is in the patient’s hand and in the neurologist’s eye

**DOI:** 10.1007/s10072-024-07626-1

**Published:** 2024-06-04

**Authors:** Alessandro Bertini, Sveva Lenti, Giorgia Libelli, Riccardo Ronco, Serena Oliveri, Kora Montemagno, Alberto Priori, Tommaso Bocci

**Affiliations:** 1 “Aldo Ravelli” Center for Neurotechnology and Experimental Brain Therapeutics, Department of Health Sciences, University of Milan, Via Antonio di Rudinì 8, 20142 Milan, Italy; 2 Clinical Neurology Unit, Department of Health Sciences, Azienda Socio-Sanitaria Territoriale Santi Paolo E Carlo, University of Milan, Via Antonio di Rudinì 8, 20142 Milan, Italy; 3 Neurology Unit, Department of Medicine and Surgery, University of Parma, Via Gramsci 14, 43126 Parma, Italy

**Keywords:** Neurological diagnosis, Thalamic hand, Parietal hand, Pseudoperipheral hands, Split hand, Alien hand

## Abstract

The objective of this study was to encompass current knowledge about pathophysiological mechanisms of those specific hand postures or deformities caused by central nervous system disorders. In the era of high-resolution neuroimaging and molecular biology, clinicians are progressively losing confidence with neurological examination. Careful hand observation is of key importance in order to differentiate neurological from non-neurological conditions, central from peripheral aetiologies, and organic from functional disorders. Localizing the potential anatomical site is essential to properly conduct subsequent exams. We provided a practical guide for clinicians to recognize hand patterns caused by central nervous system disorders, avoiding mimicking conditions, thus optimizing and prompting the diagnostic pathway.

## Introduction

A number of central neurological syndromes are often characterized by typical hand postures, which are sometimes pathognomonic, usually pointing to a precise topographic localization of the lesion. Nonetheless, these features are underestimated, and their early diagnostic value is not clearly recognized. Every experienced neurologist should carefully observe patient hands as soon as the patient comes to the doctor’s attention; that allows to optimize the diagnostic approach, thus avoiding any useless instrumental examination. This approach is of key importance in order to differentiate neurological from non-neurological conditions, as occurs for the so-called “striatal hand”, often misdiagnosed with hand deformities secondary to osteoarthrosis or rheumatoid arthritis. In other cases, their recognition raises the suspicion of low-progression diseases, potentially treatable if diagnosed at an early stage, thus driving further second-line investigations: this is the case of both “thalamic hand” and “parietal hand”, usually associated with low-grade gliomas. Several clinical clues are paramount in differentiating central from peripheral etiology, as in the case of pseudoperipheral hand palsies, prompting specific therapy in an emergency setting (e.g. revascularization therapy). Finally, temporal seizures are typically characterized by particular hand postures, suggesting their exact cortical origin and drive neurophysiological examinations, including high-density EEG, in order to improve the planning of a future intra-operative monitoring, as occurs for refractory temporal epilepsies. In this narrative review, we encompass current knowledge about “central neurological hands”, their incidence, pathophysiology and differential diagnoses; we then provide a practical guide for clinicians to recognize these features strengthening the importance of an early diagnosis aimed at modifying the disease course. A final paragraph will deal with the psychopathological dystonias, a rare psychogenic disorder often misdiagnosed with organic causes.

## Methods

Research strategies included screening literature on PubMed and Google Scholar databases updated until 1th September 2023. The search keywords were (“hand” or “hand postures”) AND (“parkinson” or “parkinsonism” or “parietal” or “pseudoperipheral” or “thalamic” or “epilepsy” or “psychogenic”). Two authors (A.B. and S.L.) screened records of search outputs for pertinence to the topic and English language only (Table 1).

**Table 1:** Central hands

Central hands	Key features	Main causes	Differential diagnosis
Split hand	- Tenar eminence more involved than hypotenar eminence - Atrophy does not respect nerve trunk or root territories	-Amyotrophic lateral sclerosis -Spinal muscular atrophy -Kennedy’s disease -Post-polio syndrome -Charcot-Marie-Tooth disease -Spinocerebellar ataxia type-3	Ulnar/Median nerve lesion
Pseudoperipheral hand	- Relative sparing of sensibility as compared to motor involvement - Preserved synkinetic wrist extension following fist closure (pseudoradial palsy) - presence of a gradient of weakness between the radial side and the ulnar side	-Stroke (< 1% of all ischemic strokes), mainly embolic	Ulnar/Median/Radial nerve lesion: synkinetic movements not preserved, motor and sensory symptoms strictly limited to affected fingers
Alien hand	- *Frontal variant* • Affected hand: (right) dominant • Awareness: preserved • Main feature: impulsive groping, compulsive manipulation of objects, and difficulty releasing objects when grasped • Associated features: leg/arm weakness, non-fluent speech, frontal syndrome - *Callosal variant* • Affected hand: (left) non-dominant hand • Awareness: absent • Main feature: intermanual conflict • Associated features: apraxia, tactile and visual anomia, agraphia, alexia and neglect - *Parietal variant* • Affected hand: mainly (left) non-dominant hand • Awareness: absent-reduced • Main feature: limb levitation, ataxia, purposeless or non-conflicting movements • Associated features: visual or sensory neglect, body schema dysfunction, hemiasomatognosia, spatial neglect, or astereognosia	-*Frontal variant *→ stroke in the anterior communicating artery territory, lesioning the dominant (left) medial prefrontal cortex/supplementary motor cortex (± involvement of the corpus callosum) -*Callosal variant *→ isolated corpus callosum injury (e.g. cancer, stroke) -*Parietal variant *→ corticobasal syndrome (subacute, unilateral/bilateral); Creutzfeldt-Jakob disease; stroke in the parietal lobe (acute, unilateral)	-Hemiballismus, focal dystonia, task-specific limb dystonia, pseudoathetosis (abnormal movement with preserved awareness) -Hemineglect (loss of awareness without abnormal movements)
Talamic hand	- *Jerky dystonic unsteady hand* • Hyperkinetic disorder presenting with myoclonus, dystonia, rubral tremor or choreoathetosis • Hand is usually flexed and pronated, with the thumb buried beneath the other fingers • Delayed onset after lesion - *Pseudochoreoathetotic hand* • Piano-playing movements of the fingers on an outstretched hand and is associated • Profund loss of proprioception • Aggravated by closing the eyes - *Pure hyperkinetic movements* (focal dystonia, asterixis, athetosis and tremor): very rare	- *Jerky dystonic unsteady hand* → pulvinar nucleus lesion - *Pseudochoreoathetotic hand *→ posterior limb of internal capsule + ventral posterolateral nuclei of the thalamus lesion	Other causes of tremor, myoclonus, dystonia, choreoathetosis, asterixis
Striatal hand	- Fixed metacarpophalangeal flexion, interphalangeal (hyper)extension and distal phalangeal subluxation/distal interphalangeal flexion, global ulnar hand deviation - Do not worsen with activity - unrelated to the wearing-off process - Fixed - Frequently unilateral - Persist during sleep - Not associated with tremor	-Parkinson disease (advanced > early stage) -Parkinsonisms (rare)	-Hand dystonia: not fixed, worsen with activity, does not persists during sleep, associated with tremor -Rheumatoid arthritis: pain and swelling in joints, more frequently bilateral
Epileptic hand (temporal lobe)	- Manipulative automatisms have low lateralizing value (except for RINCH with high lateralizing value, controlateral to the epileptogenic zone) - Non-manipulative automatisms have high lateralizing value (controlateral to the epileptogenic zone) - Dystonic postures have high lateralizing value (controlateral to the epileptogenic zone)	-Temporal lobe epilepsy	

## Central hands

### Split hand

The split hand syndrome is a dissociated pattern of atrophy in hand muscles, clinically characterized by a severe wasting observed in the thenar eminence, namely the abductor pollicis brevis (APB) and first dorsal interosseous muscle (FDI) muscles, with relative sparing of the abductor digit minimi (ADM) in the hypothenar eminence [1, 2]. Split hand syndrome has been widely described in amyotrophic lateral sclerosis, affecting around 70% of patients at the time of diagnosis [3]. However, it has also been observed in other motor neuron disorders and neuropathies, comprising spinal muscular atrophy, Kennedy’s disease and post-polio syndrome, Charcot-Marie-Tooth disease, and spinocerebellar ataxia type-3 [4, 5]. Three main theories have been put forward to explain the underlying mechanisms that give rise to this pattern of muscle wasting. The first hypothesis relies on a cortical dysfunction: Menon and colleagues documented a greater degree of cortical hyperexcitability in the APB/FDI muscles, as compared with ADM [3, 6]. The second theory posits that the split hand is caused by a dysfunction in the axonal membrane channels, which are responsible for the transmission of electrical signals along the motor neurons driving muscle movement [7]. Indeed, studies have shown that motor axons in the affected muscles have more prominent persistent sodium currents in APB/FDI as compared to ADM, leading to a higher axonal excitability and increased susceptibility to degeneration [8–10]. This last hypothesis is based on the different intrinsic physiological variability at the neuromuscular junction of small hand muscle. Accordingly, repetitive nerve stimulation studies revealed that the percent of area decrement was significantly greater in APB and FDI than ADM both in ALS and healthy controls, which underlies an intrinsic poorer plaque transmission in the thenar eminence muscles [11].

### Pseudoperipheral hand

**Fig. 1:**
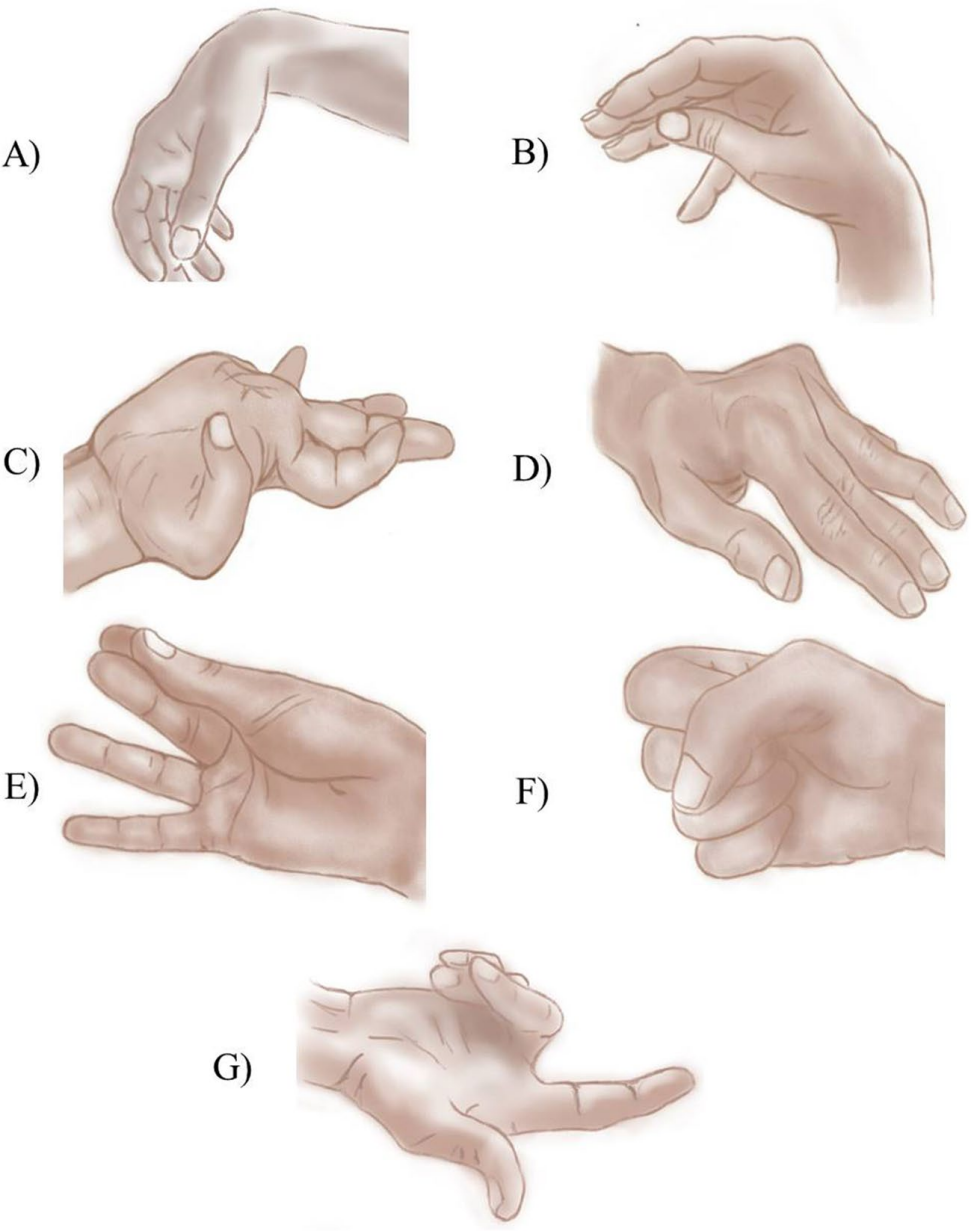
Neurological hands. Pseudoradial hand (**A**), Alien hand, posterior variant (**B**), Thalamic hand, pseudochoreoathetotic variant (**C**), Striatal hand (**D**), “Pincer” hand (**E**), “Fist” hand (**F**), Clenched fist hand (**G**)

Pseudoperipheral hand palsy defines a sudden, isolated, and focal weakness of the hand secondary to stroke (< 1% of all ischemic strokes [12]) with a radial, ulnar or median distribution mimicking peripheral palsy. Pseudoradial palsy (Fig. 1A) is the most common pattern, characterized by hand drop with the inability to extend both wrist and fingers. The etiology is usually cardioembolic [12] with the cortical lesion localized in the omega-shaped “hand knob” motor area, in the precentral gyrus, immediately after the intersection of the superior frontal and precentral sulcus [13, 14]. Unfortunately, pseudoperipheral palsies are often misdiagnosed and confused with the more frequent peripheral palsy, which precludes revascularization therapies if warranted, as well as secondary prevention treatments. However, these few clues can guide diagnosis toward central etiology: (i) the (relative) sparing of sensibility, (ii, for pseudoradial palsy) the preserved synkinetic wrist extension following fist closure [15], (iii, for pseudoradial and pseudoulnar palsy) the presence of a gradient of weakness between the radial side and the ulnar side (and vice-versa) rather than motor deficits limited strictly to one or a few fingers [13]. Indeed, finger movements are cortically controlled by a highly distributed network rather than by functionally and spatially discrete groups of neurons controlling each finger [13].

### Alien hand and parietal hands

Alien hand syndrome (AHS) is a rare neurological disorder characterized by involuntary upper limb movements associated with a sense of loss of limb ownership [16]. Three main variants of AHS are recognized: frontal, callosal and posterior. The frontal variant affects the dominant (right) hand and is characterized by impulsive groping, compulsive manipulation of objects, and difficulty in releasing objects when grasped [17, 18]. Awareness is preserved, but patients are unable to voluntarily suppress intrusive movements [19]. Additional features include frontal lobe signs, such as leg or arm weakness, non-fluent speech, grasp reflex, apathy, disinhibition, or personality alterations. It is most commonly secondary to stroke in the anterior communicating artery territory, lesioning the dominant (left) medial prefrontal cortex and supplementary motor cortex with or without involvement of the corpus callosum [18, 20]. The callosal form, caused by isolated corpus callosum injury [21, 22], exclusively affects the non-dominant (left hand) in right-handed patients. It is characterized by inter-manual conflict, with minimal limb weakness and absence of frontal features [16], even though mixed frontal and callosal AHS due to mesial frontal and extensive callosal lesions have been reported [23]. Patients usually present with apraxia, tactile and visual anomia, agraphia, alexia and neglect [24]. The posterior variant (Fig. 1B) is characterized by strong feelings of estrangement from the affected limb, less complex motor activity (e.g., limb levitation, ataxia, non-purposeful or non-conflicting movements), and parietal sensory deficits (visual or sensory neglect, body schema dysfunction, hemiasomatognosia, spatial neglect, or astereognosis). The non-dominant (left) hand is frequently affected due to a lesion in the non-dominant (right) parietal lobe (posterior postcentral gyrus, posterior primary sensory cortex, and tertiary somatosensory cortex in the superior parietal lobule) [25]. However, lesions of the anterior part of the parietal cortex may present with different features, namely those of a “grasping” and a “repulsion” hand [26]. Common etiologies comprise neurodegenerative disorder (especially tauopathies), including Cortico-Basal Syndrome (CBS), slowly evolving and low-grade tumors of the parieto-occipital cortex, Creutzfeldt-Jakob Disease (CJD), and stroke of the parietal lobe or posterior cerebral artery territory [27]. Alien hand secondary to neurodegenerative disorders usually has an insidious/subacute onset and tends to become bilateral as the disease progresses [16].

The differential diagnosis includes disorders characterized by involuntary movements with preserved awareness, such as hemiballismus, focal dystonia, task-specific limb dystonia, pseudoathetosis, and disorders characterized by loss of awareness without abnormal movements (e.g. hemineglect).

### Thalamic hand

Thalamic hand encompasses a group of complex abnormal postures and hyperkinetic movement disorders of the upper limb following a contralateral thalamic lesion [28–30]. Prevalence remains unknown. Associated features vary from sensory loss, pain, loss of proprioception and weakness in the affected limb. Classically, there are two main clinical phenotypes, always overlapping: a dystonic presentation and a choreoathetotic variant. The jerky dystonic unsteady hand [31] is a delayed hyperkinetic disorder presenting with myoclonus, dystonia, rubral tremor or choreoathetotic movements in a mixed complex presentation, secondary to infarcts in posterior choroidal territory involving the posterior area of the thalamus (pulvinar nucleus), thus sparing other thalamic, subthalamic and midbrain structures. The hand is usually flexed and pronated, with the thumb buried beneath the other fingers [32]. Conversely, pseudochoreoathetotic hand (Fig. 1C) is characterized by piano-playing movements of the fingers on an outstretched hand and is associated with profound loss of proprioception [33]. Other hyperkinetic disorders such as pure focal dystonia, asterixis, athetosis and tremor, affecting hand, have rarely been reported in literature [34].

### Striatal hand

Striatal hand deformities are abnormal hand postures that occur in up to 10% of patients with advanced Parkinson’s disease (PD), even though they can be observed in the early stages of PD and in other parkinsonisms as well [35, 36]. Clinically, the striatal hand deformity (Fig. 1D) is characterized by fixed metacarpophalangeal flexion, interphalangeal (hyper)extension and distal phalangeal subluxation, leading to distal interphalangeal flexion, and global ulnar hand deviation [37]. The pathogenesis is not fully understood, but is likely related to a combination of parkinsonian features such as rigidity [38] and hypertonia, leading to fixed contractures, reduction of sarcomere length [39], structural musculoskeletal changes, and loss of postural reflexes. Important risk factors include disease duration, amount of rigidity, female sex, and dopamine agonist consumption [40], due to an imbalance between decreased dopaminergic level and increased GABAergic and cholinergic levels [41].

Striatal hand deformities must be differentiated from hand dystonia which occurs in primary dystonic state, parkinsonism syndromes, and PD as a complication of pharmacotherapy. This phenomenon correlates with a younger age at disease onset, but not with dyskinesia, levodopa equivalent dose, or the severity of cognitive dysfunction [42, 43]. Unlike dystonia, as observed for the writer’s or pianist’s cramp, striatal hand deformity is commonly (i) unrelated to activity, (ii) unrelated to the wearing-off phases, (iii) fixed, (iv) persistent during sleep, and (v) not associated with tremor [37, 44, 45]. Furthermore, striatal hand deformity is often misdiagnosed with hand deformities secondary to rheumatoid arthritis. However, rheumatoid arthritis affects joints rather than muscles, is more frequently bilateral as compared to striatal deformities which are usually lateralized to the most affected limb, and is associated with inflammation, pain, and swelling [46]. An early differential diagnosis is crucial for a possible infiltrative therapy with botulinum, which has been described to improve striatal hand movements [35, 47].

### Temporal lobe epileptic hands

Surgery of epilepsy is an important treatment option in patients with focal epilepsy, which is notoriously refractory to medical treatment in about 30% of cases [48]. For purpose of planning surgery, despite the advent of more sophisticated techniques, the study of ictal semiology is paramount in the process of localization of epileptogenic focus. This becomes particularly important in focal epilepsies with unremarkable MRI or scalp EEG [49, 50]. Hand semiology is an important, immediate, and costless localizing toll in epilepsy, pointing at temporal lobe and, more rarely, frontal lobe etiology. Ictal hand evaluation should focus on the presence of (i) manual automatisms and (ii) dystonic hand postures, and (iii) the pattern of dystonic hand posture.

Ictal manual automatisms encompass heterogeneous stereotyped, non-purposeful/semi-purposeful repetitive movements [51] occurring in more than 80% of temporal lobe epilepsies (TLE) [52], with a different lateralizing value, depending on their pattern. Manipulative automatisms, such as picking at bedclothes, repetitive movements of fingers, and fumbling, are semi-purposeful and arrhythmic hand automatisms, with low laterality value [52, 53]. However, when occurring in association with contralateral dystonic hand posture, their lateralizing value is strong, namely ipsilateral in 85% of cases [52, 54]. Conversely, non-manipulative automatisms (e.g. repetitive raising and lowering of upper extremity with a circulatory component resembling waving, flaunting or circling), which are typically purposeless and rhythmic, are a reliable lateralizing sign, contralateral to the hemisphere involved in TLE [53], regardless the presence of dystonia. Interestingly, in 2006, Lee and colleagues [55] firstly described a group of manipulative automatisms, the so-called rhythmic ictal non-clonic hand (RINCH) motions, which, as compared to classic manipulative automatisms, occur contralateral to seizure onset (in 93% of cases) [56]. The most common RINCH motions are in the decreasing order: hand opening/closing, finger rubbing, milking motions, finger flexion/extension, and pill rolling [56]. Interestingly, both non-manipulative automatisms and RINCH motions can be associated with dystonic hand posturing or precede them, or, as proposed by Keleman and colleagues [53], may belong to the clinical spectrum of dystonia, namely “dystonic rhythmic automatisms”. Therefore, hand dystonia, both presenting as fixed postures and “dystonic rhythmic automatisms”, is the most reliable lateralizing sign in TLE, occurring contralateral to the onset of seizures, independently to the pattern of hand automatism: manipulative, non-manipulative or RINCH motions. The typical fixed hand posture shows flexion of the wrist and metacarpophalangeal joints, extension of the fingers, as well as rotation in the forearm [54]. Specifically, Ferando and colleagues [57] identified three patterns of hand posture which highly predict temporal lobe localization: the “cup”, “politician’s fist” and “pincer” (Fig. 1E) which appear identical in the position of thumb (flexed), index (extended) and middle finger (flexed); conversely, the “fist” (Fig. 1F) and “pointing postures” are related to frontal lobe epilepsy.

## Psychogenic hand disorders

Psychogenic hand disorders can be categorized into four distinct groups: (i) factitious wound creation and manipulation; (ii) factitious edema; (iii) psychopathological dystonias; and (iv) psychopathological sensory abnormalities and psychopathological Complex Regional Pain Syndrome [58]. Discussion of categories (i), (ii), (iv) is beyond the purpose of this review.

Among psychopathological dystonias, two commonly misdiagnosed conditions are the Clenched Fist Syndrome (CFS) and the Psycho-Flexed Hand (PFH), which often resemble organic dystonia. CFS is usually characterized by the fixed flexion and sustained contraction of two–three digits on the ulnar side, with the thumb and index finger typically spared, resulting in the hand being clenched almost into a fist (Fig. 1, G) [59, 60]. On the other hand, PFH presents with a similar pattern to CFS, although the patients’ fist is not entirely clenched [61]. In PFH, the dominant hand is usually affected, whereas no preferential involvement is observed in CFS. Swelling is a consistent feature in both conditions. Attempts at passive extension of the fingers are painful; however, in certain situations, such as when patients are asleep or believe they are unobserved, they can extend their fingers without experiencing pain [60]. Full extension can be achieved under anesthesia, but the contractures reoccur immediately after regaining consciousness. In advanced cases, the contractures become permanent due to changes in the soft tissues, joints, and tendons. A positive psychiatric history, including schizophrenia and depression, is commonly associated with these conditions [60]. Moreover, it is often noted that these conditions are preceded by minor injuries or surgeries, and there is a notable discrepancy between the magnitude of the trauma and the severity of the contractures. Nerve conduction studies are paramount both in CFS and PFH diagnosis. Differential diagnosis should include rheumatologic diseases, Dupuytren contracture, hand dystonia, and peripheral nerve lesions [61].
